# Group B Streptococcus serotypes associated with different clinical syndromes: Asymptomatic carriage in pregnant women, intrauterine fetal death, and early onset disease in the newborn

**DOI:** 10.1371/journal.pone.0244450

**Published:** 2020-12-31

**Authors:** Yulia Schindler, Galia Rahav, Israel Nissan, Liora Madar-Shapiro, Julia Abtibol, Moti Ravid, Yasmin Maor

**Affiliations:** 1 Microbiology laboratory, Maayaney Hayeshua, Bney Brak, Israel; 2 The Sackler School of medicine, Tel Aviv University, Tel Aviv, Israel; 3 Infectious Disease Unit, Sheba Medical Center, Tel Hahomer, Israel; 4 National Public Health Laboratory, Ministry of Health (Israel), Tel-Aviv, Israel; 5 Infectious Disease Unit, Wolfson Medical Center, Holon, Israel; Universidade de Lisboa Faculdade de Medicina, PORTUGAL

## Abstract

**Objectives:**

To study Group B Streptococcus (GBS) isolates associated with different clinical syndromes: asymptomatic carriage in pregnant women, intrauterine fetal death (IUFD), and early onset disease (EOD) in the newborn.

**Methods:**

GBS isolates were collected from asymptomatic pregnant women admitted for labor, IUFD cases, and neonates with EOD. Serotypes and antibiotic susceptibilities were determined. Multilocus sequence typing (MLST) was performed to assess genetic epidemiology.

**Results:**

GBS carriage rate was 26.1% (280/1074). The dominant serotype among asymptomatic pregnant women was VI [98/240 women (40.8%)], followed by serotypes III, V and IV in 42/240 (17.5%), 30/240 (12.5%) and 28/240 (11.7%) women, respectively. The dominant serotype in IUFD cases was serotype VI [10/13 (76.9%)]. In contrast the prevalent serotype among EOD cases was III [16/19 (84.2%)]. ST-1 was associated with IUFD [7/13 (53.8%)], ST-17 was associated with serotype III and EOD in the newborn 14/19 (73.7%)]. Erythromycin and clindamycin resistance reached 36.8%, 7.7% and 20.0%among EOD, vaginal carriage and IUFD, respectively.

**Conclusions:**

Serotypes VI and ST-1 were dominant among asymptomatic pregnant women and in IUFD cases while EOD was associated with serotype III and ST-17. Invasive mechanisms thus may differ between IUFD and EOD in the newborn and virulence may be related to capsule serotype. Resistance rates to erythromycin and clindamycin were high in EOD cases.

## Introduction

Group B *Streptococcus* (GBS) is a leading cause of neonatal sepsis and meningitis as well as amnionitis and sepsis [1, 2]. Two GBS-associated syndromes are described in neonates: early-onset disease (EOD) which is responsible for 40% of cases, and late-onset disease (LOD) associated with 60% of cases. EOD occurs in the first week of life (0–6 days) and is associated mainly with bacteremia [2, 3]. EOD results from vertical transmission of the bacteria through contaminated amniotic or vaginal secretions of a colonized mother to her neonate during, or just before delivery [1]. LOD occurs after the first week of life and up to the age of 3 months and is characterized by a high rate of meningitis [2, 3]. LOD is not necessarily associated with GBS carriage in the mother, and therefore is not attenuated by preventive strategies such as screening and antibiotic treatment of the pregnant mother. Approximately 10% of the infected babies die from GBS infection and 20% of survivors suffer permanent handicap.

The overall incidence of EOD in the newborn is between 0.3 and 0.6 infants per 1,000 live births [1–3] and it varies geographically. The incidence of GBS colonization in Israeli pregnant women is 21.6% and the incidence of EOD is low with only 0.27 cases per 1,000 live births [4]. Therefore, the Israeli Ministry of Health guidelines (22/2005) do not advocate universal GBS screening during pregnancy, but instead promote antibiotic prophylaxis prior to delivery according to risk factors.

GBS during pregnancy can also ascend from the vagina into the uterus and cause infection of the amniotic fluid and the fetus (amnionitis). This is associated with fetal injury, preterm birth, stillbirth, and endometritis [5, 6]. There is limited data regarding the incidence of intrauterine GBS infection worldwide [6].

GBS strains can be divided into 10 capsular serotypes (Ia, Ib, and II to IX) [7–9]. There are more than 750 sequence types (STs) defined by multilocus sequence typing (MLST). Most human isolates cluster into six clonal complexes (CCs) [8, 9]. Serotype III is associated with neonatal meningitis, and is responsible worldwide for a substantial proportion of EOD cases (40–60%) and the majority of LOD cases (60–80%) [2, 3, 5]. Studies showed that one particular hypervirulent GBS genotype (ST-17), represents 70% of GBS isolates responsible for neonatal meningitis and belongs exclusively to serotype III [7–9].

In past it was assumed that all GBS isolates are highly susceptible to penicillin [10]. Therefore, the drug of choice for treating GBS colonization in pregnant women is penicillin. For GBS-colonized mothers with β-lactam allergy, erythromycin or clindamycin are used [1–3]. Recent data reports increasing rates of macrolide resistance [10, 11]. Also, several recent studies demonstrated a worrying increase in GBS isolates with resistance to penicillin [12, 13]. In Israel, screening does not include antibiotic susceptibility and there is no data regarding GBS resistance rates. Women with β-lactam allergy may thus receive inappropriate treatment. Also, in rare cases of resistance to penicillin treatment may be inappropriate.

Maayaney Hayeshua Medical Centre (MHMC), in Bney Brak, Israel, serves mainly an Orthodox Jewish community. In 2017, 11,967 newborns were delivered. The incidence of neonatal EOD disease in MHMC increased significantly from 0.25/1,000 during 2016 (3 cases) to 0.51/1000 live births (7 cases) in 2017, significantly higher than the national Israeli reported rate of 0.27/1,000 live births [4]. Furthermore, 17 pregnant women during 2017 had intrauterine fetal death (IUFD) after the 20th week of gestation. Seven of these were associated with GBS amnionitis, increasing the incidence of stillbirth due to GBS to 0.58 per 1000 live births. These high rates led us to begin as of August 2017 GBS screening in pregnant women admitted for labor.

The aim of our study was to describe the molecular characteristics and antibiotic susceptibilities of GBS isolates according to different clinical syndromes: a) Asymptomatic carriage in pregnant women, b) IUFD, and c) EOD in the newborn.

## Material and methods

### Study population

We studied a convenience sample of asymptomatic pregnant women admitted for labor during 2017 to MHMC7. Women were screened for GBS carriage using recto-vaginal swabs, as part of the universal screening implemented in MHMC from August 2017 for all pregnant women admitted for labor regardless of gestational age or for pregnant women with miscarriage in the third trimester or amnionitis. Some women were screened in the community and were not included in this analysis. Implementation of screening women on admission to labor was slow. We eventually had samples from 1,074 women from the 1^st^ of August to 31 December 2017, 22% of all women giving birth in the hospital during this period.

280 women had positive GBS cultures, forty isolates were not frozen for further study. Thus 240 isolates were available for further investigation. We also included all GBS isolates obtained from IUFD remains and isolates from EOD in newborns during 2013–2017.

The study was approved by the ethics committee of Maayaney Hayeshua Medical Centre (approval number 0023-18-MHMC). Data was summarized from patients files regarding age, gravidity, parity, gestational age, mode of delivery, weight at birth and pregnancy outcome (alive, dead, EOD).

### Serotyping

GBS isolates were preserved in sterile Brain Heart Infusion broth with 15% glycerol (HyLabs, Israel) at −70°C for long-term storage and at 4°C for short-term maintenance. Frozen GBS strains were revived; twice sub cultured and incubated. One to three colonies of overnight GBS cultures were suspended in one ml of saline and heated for 10 minutes at 100°C in a dry bath. Four hundred μl of lysed bacteria were transferred for DNA extraction [EZ1 Virus Mini kit (Qiagen) or manual extraction using the Bacterial kit (Presto, Geneaid) and lysis buffer (Lysozyme, Geneaid)]. Extracted DNA was stored at −20°C. Amplification was performed using specific primers for every serotype and a fluorescent probe labeled with 6-FAM [14]. Positive and negative controls were included in every run.

### MLST analysis

Primer sets corresponding to seven housekeeping genes (*adhP*, *atr*, *glcK*, *glnA*, *pheS*, *sdhA*, *and tkt)* were constructed with reference to the MLST website (http://pubmlst.org/sagalactiae/), with alleles and sequence type (ST) using the *S*. *agalactiae* MLST database. The relationships of each ST were analyzed by eBURST version 3.1 (http://eburst.mlst.net/v3/mlst_datasets/).

### Antibiotic susceptibility testing

Antibiotic susceptibility testing to penicillin, clindamycin and erythromycin was performed using disk diffusion according to the Clinical and Laboratory Standards Institute (CLSI) guidelines [15]. Isolates sensitive to clindamycin, but resistant to erythromycin, were further tested using the D-zone test to determine inducible clindamycin resistance.

### Statistical methods

Statistical analyses were done using SPSS version 24.0 (SPSS Inc., Chicago, IL, USA). Prevalence was calculated based on positive GBS results within the study population. Comparison of proportions of GBS colonization in MHMC and in pregnant women from the general pregnant woman population on Israel was based on national data [4]). Comparisons were performed between women with and without asymptomatic GBS carriage. Groups were compared according to GBS status and clinical presentation: asymptomatic carriage, IUFD and EOD. Chi-square for categorial variables and student T test for continuous variables were used. When appropriate Fisher exact test was used. Significance was set at p <0.05.

## Results

### GBS colonization

One thousand and seventy-four asymptomatic pregnant women in labor were screened for GBS. Two hundred and eighty were positive for GBS [(26.1%) 95% confidence interval (CI), 23.4% to 28.6%]. GBS colonization rate in MHMC was significantly higher than the overall prevalence of 21.6% reported among pregnant Israeli women (p<0.0001) [4]. Mean age was 28.9 years [standard deviation (SD) 5.9), mean gravidity was 4.3 (SD 3.2), mean parity was 3.0 (SD 2.5), mean gestational age was 39.3 weeks (SD = 2.0) and mean birth weight was 3299.5 g (SD 506.1). Baseline characteristics including age, gestational age, gravidity, parity, , , and birth weight were similar between GBS-positive and GBS-negative women (Table 1). There were no demographic differences between women with asymptomatic carriage, women who gave birth to neonates with EOD or women who suffered from IUFD.

**Table 1:** Characteristics of pregnant women during labor with positive and negative GBS rectovaginal cultures.

Variable	Women with positive GBS cultures	Women with negative GBS cultures	p- value
	280 (26.1%)	794 (73.9%)	
**Maternal age**			0.054
** < 30 years**	158 (56.4)	467 (58.8)	
** 30–40 years**	105 (37.5)	304 (38.3)	
** > 40 years**	17 (6.1)	23 (2.9)	
**Gestational age**			0.192
** < 34 weeks**	4 (1.4)	10 (1.3)	
** 34–37 wee1ks**	40 (14.3)	82 (10.3)	
** >37 weeks**	236 (84.3)	702 (88.4)	
**Gravidity**			0.463
** ≤3**	137 (48.9)	410 (51.6)	
** -8**	114 (40.7%)	291 (36.6)	
** >8**	29 (10.4)	93 (11.7)	
**Parity**			0.311
** ≤ 3**	177 (63.2)	540 (68.0)	
** 4–8**	89 (31.8)	215 (27.1)	
** ≥ 8**	14 (5.0)	39 (4.9)	
Birth weight*			0.275
** < 2,500 g**	21 (7.5)	36 (4.9)	
** 2,500–4,000 g**	238 (85.0)	642 (87.8)	
** >4,000 g**	21 (7.5)	53 (7.3)	

Characteristics of pregnant women during labor with positive and negative

GBS rectovaginal cultures. Results are presented as n (%), unless otherwise stated.

*Birth weight was available for 1011 newborns.

### Serotype distribution

**Fig 1:**
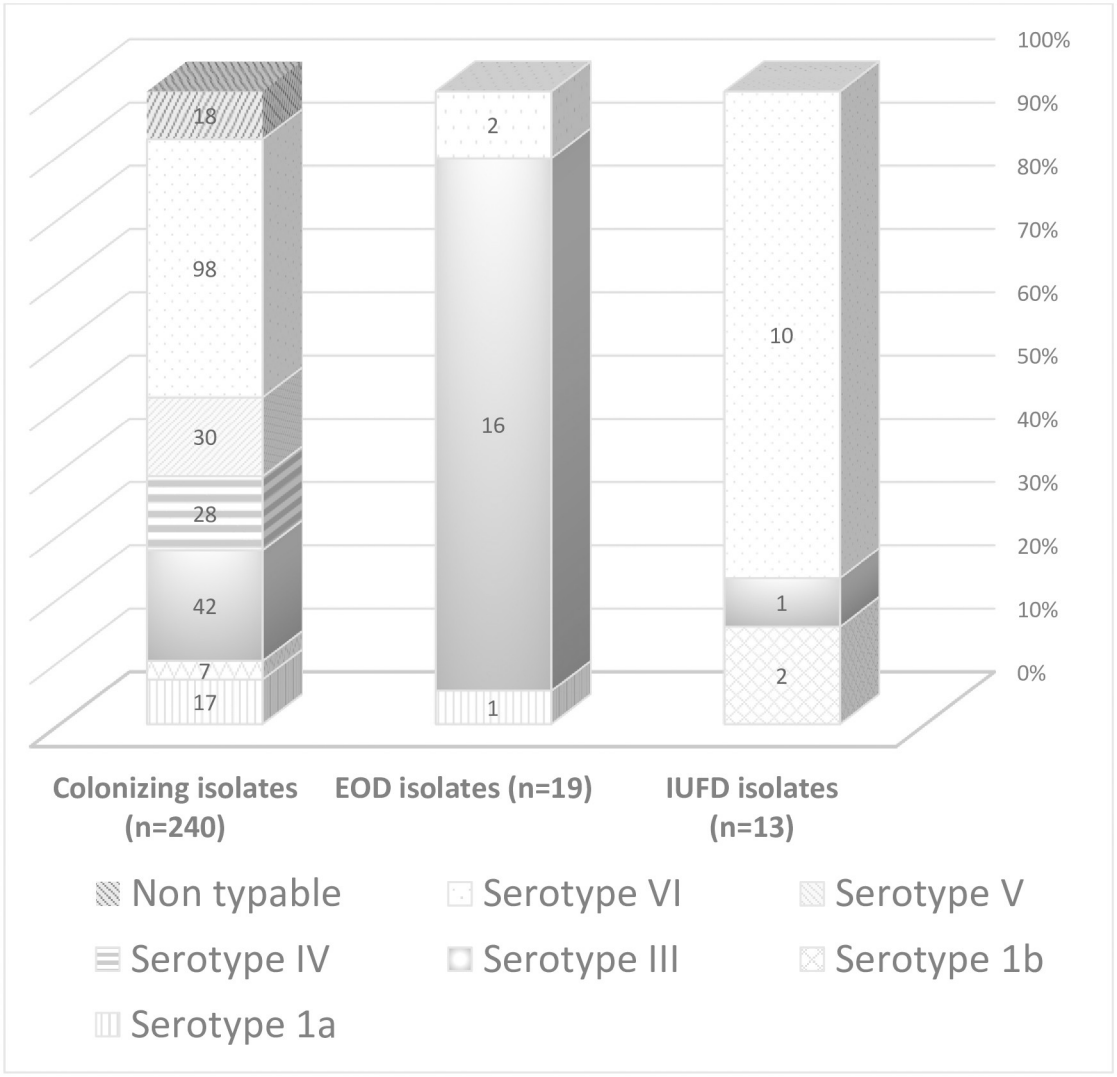
Distribution of serotypes among GBS isolates recovered from colonized pregnant women, from neonates with early onset disease (EOD) and from intra uterine fetal death (IUFD) cases. Colonizing isolates—vaginal isolates from colonized pregnant women. EOD isolates—isolates from blood cultures of neonates with EOD. IUFD isolates—isolates from cultures of IUFD remains.

Among 240 GBS isolates from asymptomatic pregnant women available for analysis the dominant serotype was VI [[98/240 isolates (40.8%)]], followed by serotypes III, V, IV, 1a, and 1b [42/240 isolates (17.5%), 30/240 isolates (12.5%), 28/240 isolates (11.7%), 17/240 isolates (7.1%), and 7/240 isolates (2.9%), respectively]. Eighteen of 240 isolates (7.5%) were non-typable. The dominant serotype among 13 GBS isolates from IUFD cases was VI [10/13 isolates (76.9%)], In contrast, serotype distribution of 19 GBS isolates from neonates with EOD revealed that serotype III dominated [16/19 (84.2%] (Fig 1).

### Correlation between capsular polysaccharide serotypes, and genotypes (ST type)

The genetic diversity of 32 isolates from asymptomatic women selected randomly, 13 IUFD isolates, and 19 EOD isolates were analyzed using MLST. ST-1 was the most common ST type among 13/32 isolates from asymptomatic women (40.6%) and 7/13 IUFD isolates (53.8%) and was associated with serotype VI in 20 of 21 cases (95.2%). ST-17 was identified in 9/32 isolates from asymptomatic women (28.1%), 1/13 IUFD isolates (7.7%) and 14/19 EOD isolates (73.7%) and was associated with serotype III in 21/24 cases (95.8) (Table 2).

**Table 2:** Correlation between GBS sequence type and clinical syndrome.

sequence type	Clinical syndrome		
	Colonization isolates n = 32 (%)	IUFD isolates n = 13 (%)	EOD isolates (n = 19)
ST-1	13 (40.6)	7 (53.8)	1 (5.3)
ST- 4	0	1 (7.7)	0
ST-6	0	1 (7.7)	0
ST-8	4 (12.5)	0	0
ST-12	1 (3.1)	0	0
ST-17	9 (28.1)	1(7.7)	14 (73.7)
ST-19	4 (12.5)	1 (7.7)	0
ST-23	0	0	1 (5.3)
ST-27	0	1 (7.7)	2 (10.5)
ST-106	0	0	1 (5.3)
ST-196	0	1 (7.7)	0
ST-459	1 (3.1)	0	0

Correlation between GBS sequence type and clinical syndrome colonizing strains (rectovaginal cultures), EOD strains (blood cultures of neonates with EOD) and IUFD strains (fetal remains cultures).

EOD—early onset disease; IUFD—intrauterine fetal death

The relations between serotype, genotypes and clinical syndrome are shown in Table 3. ST-1 related to serotype VI in 20 of 21 cases (95.2%)and was associated with colonization and IUFD and vaginal colonization, and ST-17 was related to serotype III in 2 of 24 cases (87.5%) and was associated with EOD.

**Table 3:** Distribution of GBS isolates according to ST-type and serotype.

ST type	Serotype III	Serotype IV	Serotype VI	Serotype 1b	Serotype 1a	Total
**ST1**	1	0	20	0	0	21
**ST4**	0	0	0	1	0	1
**ST6**	0	0	0	1	0	1
**ST8**	0	0	4	0	0	4
**ST12**	1	0	0	0	0	1
**ST17**	21	0	2	0	1	24
**ST19**	4	0	1	0	0	5
**ST23**	1	0	0	0	0	1
**ST27**	2	0	1	0	0	3
**ST196**	0	0	1	0	0	1
**ST459**	0	1	0	0	0	1
**Total isolates tested**	31	1	29	2	1	64

Distribution of GBS isolates according to ST-type and serotype. Isolates were from asymptomatic pregnant women, IUFD cases and isolates from neonates with EOD.

EOD—early onset disease; IUFD—intrauterine fetal death

### Antimicrobial susceptibility

All GBS isolates tested [vaginal carriage (n = 280), IUFD (n = 13) and EOD (n = 19)] were susceptible to penicillin. Fifty-eight (20.7%) isolates from asymptomatic pregnant women were resistant to erythromycin and 54 (19.3%) were resistant to clindamycin. One (7.7%) IUFD isolate was resistant to both antibiotics and seven (36.8%) EOD isolates were resistant to erythromycin and clindamycin. None of the resistant isolates showed inducible clindamycin resistance (D-test phenotype).

## Discussion

We found a high rate of rectovaginal colonization in women in a single institution in Israel and found a higher prevalence of serotype VI in cases with IUFD, and serotype III in cases with EOD.

The strong association we report between clinical presentation, serotype and ST type may suggest that different serotypes and ST types are related to different virulence mechanisms and that different virulent factors are associated with IUFD and EOD. EOD has been linked by other authors to serotype III and ST-17 and is associated with both EOD and LOD worldwide, and for the vast majorities of meningitis in neonates [2, 3, 5]. The pathogenicity of this clone is associated with the presence of two specific adhesins [16]. The most frequently identified ST among colonizing GBS strains, was ST-1, which was particularly associated with serotype VI. Little is known regarding serotypes and ST types and their association with IUFD and the virulence mechanisms leading to this presentation.

In the US and Europe, in contrast to the epidemiology we report, the common colonizing serotypes are V, Ia, and II [17–19]. Serotype VI is highly prevalent among pregnant women in Japan [20, 21] and Malaysia [22]. In Taiwan, serotype VI was the second most common serotype in adults with GBS associated skin and soft tissue infections [21]. In Egypt, serotype VI was also detected as the most common colonizing serotype in pregnant women [22]. Serotype III was the predominant serotype causing EOD, in accordance with the Israeli reports, and previous studies from Europe and US [3, 4]. It seems that that the epidemiology we report is more like that reported in the middle east and Asia and less similar to the epidemiology of GBS in Europe and the US.

MLST of global GBS collections identified ST-1 and ST-19 to be significantly associated with asymptomatic colonization [7, 8]. Another study from Japan [21], reported that ST-1, and ST-19, were the main colonizers in pregnant women, like our results, and identified them as being admirably adapted to the vaginal mucosa with a poor invasion ability.

According to another Israeli study that examined colonizing GBS isolates from a low-incidence region, there was heterogeneity in ST types [23]. ST-1 was the most prevalent, and its members expressed various serotypes, with serotype V being the most common. Serotype VI was not identified in any of the samples in that study. Thus, the epidemiology we report may be unique to MHMC or may reflect a shift in GBS epidemiology in Israel, Further study in additional centers in Israel is warranted to clarify this issue.

Increased rates of GBS EOD (0.51/1000 live births) and IUFD (3.79/1000 live births) in MHMC during 2017 led us to enhanced screening of pregnant woman before labor. We found that GBS carriage rates in MHMC were higher than the carriage rate reported in Israel (26.1% versus 6–21%, respectively) [4, 24]. MHMC caters mostly Orthodox Jewish women. It is known that the prevalence of GBS colonization varies among different ethnic populations. In Netherland, African women were at a higher risk (29%, RR 1.4, CI 1.1–1.7) and Asian women were at lower risk (13%, RR 0.6, CI 0.4–0.8) for GBS carriage [25]. A recent systematic review and meta-analyses found lower prevalence of GBS carriage in pregnant women from Southern (12.5%) and Eastern Asia (11%) compared to the worldwide maternal colonization rate of 18% [26]. Another study performed in northern Israel demonstrated higher carriage rates among Arab pregnant women (19%) than among Jewish women (13.7%), p = 0.038. [27]. A previous study demonstrated a significantly higher GBS carriage rate in Orthodox non pregnant Jewish women compared with secular Jewish non pregnant women (20.6% vs. 12.8% respectively) [28]. Orthodox Jews usually live in closed, small living areas [27, 29]. Increased pharyngeal carriage rates of group A *Streptococcus* were detected in Orthodox Jews in London compared to the general population [29]; It was accounted for by the Orthodox Jew’s different family structures of large families with many young children. In Orthodox Jews marriages are mostly within the community, and women tend to give birth to more children compared to the mean number of children in the general Israeli population (7.1 compare to 3.1, respectively, data from the Israeli Central Bureau of Statistics). A previous study in Israel tried to connect between increased rates of GBS among orthodox Jewish pregnant women and ritual immersions in a Mikveh [28]. In the Orthodox Jewish patients that MHMC caters to, the high rate of GBS carriage may be related to social or religious practices such as the Mikveh, or close living quarters.

Erythromycin and clindamycin resistance rates among our colonizing strains were 20.7% and 19.3%, respectively. However, resistance rate among invasive strains causing EOD in the newborn was 36.8%. GBS resistance rates to erythromycin and clindamycin is increasing world-wide [10]. In Canada 36% of colonizing GBS isolates were resistant to erythromycin, and 33% were resistant to clindamycin [30]. In China, 52.4% of isolates from asymptomatic pregnant women were resistant to clindamycin and 64.9% were resistant to erythromycin [31]. Reported Clindamycin resistance among GBS isolated form EOD is between 16–17% [10].

In Israel, routine antibiotic susceptibilities are not performed on GBS isolated from rectovaginal screening. The high resistance rates described by us prohibits empirical use of these antibiotics for women with penicillin allergy. The increasing reports regarding penicillin resistance [12, 13] warrant also routine susceptibility test for penicillin.

Our study has several limitations. Our sampling was partial and may not represent the entire population. The number of isolates obtained from neonates with EOD and IUFD were small. The data represent women coming to give birth in MHMC that caters mostly for an Orthodox Jewish population. We did not assess level of religiousness in our study so we may be overestimating the association between religious practices and our results. Also the results may not represent all Orthodox Jewish women. Despite these limitations the increase in EOD is not related to sampling and supports a true increase in the prevalence of GBS carriage.

In conclusion, different GBS serotypes were associated with different clinical syndromes. In our dataset serotype VI, and ST-1 were found more often in pregnant women with asymptomatic carriage of GBS and in patients with IUFD while serotype III, ST-17 was found more often in EOD. This suggests different virulence mechanisms related to GBS carriage, IUFD and EOD. Further study of this hypothesis is needed. The rate of GBS carriage in women who delivered at MHMC was higher than the rate previously reported in the country and in parallel the rate of EOD in neonates at MHMC was higher than the rate previously reported in Israel. This emphasizes the importance of universal screening f. For GBS in pregnant women. Currently health authorities in Israel do not recommend routine screening of all pregnant women and advice to screen only according to risk factors. In face of the increasing clindamycin resistance, antibiotic susceptibility testing must be performed in all GBS isolates in women with an allergy to penicillin, to ensure appropriate treatment for women with penicillin allergy.

## References

[1] Centers for Disease Control and Prevention. Prevention of perinatal Group B Streptococcal disease. MMWR 2010;59(No. RR-10):1–32. 21088663

[2] Dermer P., Lee C., Eggert J., Few B. A history of neonatal group B Streptococcus with its related morbidity and mortality rates in the United States. J Pediatr Nurs 2004;19(5):357–363 10.1016/j.pedn.2004.05.012 15614260

[3] Joubrel C., Tazi A., Six A., Dmytruk N., et al Group B Streptococcus neonatal invasive infections, France 2007–2012. Clin Microbiol Infect. 2015;21(10):910–6 10.1016/j.cmi.2015.05.039 Epub 2015 Jun 5. 26055414

[4] Israeli Ministry of Health GBS annual report 2018.

[5] Nan C., Dangor Z., Cutland C.L., Edwards M.S., Madhi S.A., Cunningtonm M.C. Maternal group B Streptococcus-related stillbirth: A systematic review. BJOG An Int J Obstet Gynaecol 2015;122(11):1437–1445 10.1111/1471-0528.13527 Epub 2015 Jul 14. 26177561

[6] Seale A.C., Blencowe H., Bianchi-Jassir F, et al Stillbirth with Group B Streptococcus disease worldwide: systematic review and meta-analyses. Clin Infect Dis 2017;65(June):S125–S132 10.1093/cid/cix585 29117322 PMC5850020

[7] Shabayek S., Spellerberg B. Group B Streptococcal colonization, molecular characteristics, and epidemiology. Front Microbiol. 2018;9:437 10.3389/fmicb.2018.00437 eCollection 2018. 29593684 PMC5861770

[8] Russell N.J., Seale A.C., et al Maternal colonization with Group B Streptococcus and serotype distribution worldwide: systematic review and meta-analyses. Clin Infect Dis. 2017;65(Nov): S100–S111 10.1093/cid/cix658 29117327 PMC5848259

[9] Melin P., Efstratiou A. Group B Streptococcal epidemiology and vaccine needs in developed countries. Vaccine 2013;31:D31–D42 10.1016/j.vaccine.2013.05.012 23973345

[10] Castor M.L., Whitney C.G., Como-Sabetti K., et al Antibiotic resistance patterns in invasive Group B Streptococcal isolates. Infect Dis Obstet Gynecol 2008:1–5 10.1155/2008/727505 Epub 2009 Feb 5. 19223967 PMC2637368

[11] Lopes E., Fernandes T. et al Increasing macrolide resistance among Streptococcus agalactiae causing invasive disease in non-pregnant adults was driven by a single capsular-transformed lineage, Portugal, 2009 to 2015. Euro Surveill 2018; 23(21). 10.2807/1560-7917.ES.2018.23.21.1700473 PMC6152215 29845930

[12] Genovese C., D’Angeli F., Di Salvatore V., Tempera G., Nicolosi D. *Streptococcus agalactiae* in pregnant women: serotype and antimicrobial susceptibility patterns over five years in Eastern Sicily (Italy). Eur J Clin Microbiol Infect Dis 2020 7 22 10.1007/s10096-020-03992-8 Online ahead of print. 32700131 PMC7669783

[13] Burcham L.R., Spencer B.L, Keeler L.R., et al Determinants of Group B Streptococcal virulence potential amongst vaginal clinical isolates from pregnant women. PloS One. 2019 12 18;14(12):e0226699 10.1371/journal.pone.0226699 eCollection 2019. 31851721 PMC6919605

[14] Breeding K.M., Ragipani B., Lee K.D., Malik M., Randis T.M., Ratner A.J. Real-time PCR-based serotyping of *Streptococcus agalactiae*. Sci Rep 2016;6:1–6 27910939 10.1038/srep38523 PMC5133537

[15] Performans standards for animicrobial susceptibility testin. Clinical and Laboratory Standards Institute (CLSI) M100, 28th ed 1 2018.

[16] Teatero S., McGeer A., Low D.E., et al Characterization of invasive group B streptococcus strains from the greater Toronto area, Canada. J Clin Microbiol 2014 5;52(5):1441–7. 10.1128/JCM.03554-13 Epub 2014 Feb 19. 24554752 PMC3993709

[17] Bergal A., Loucif L., Benouareth D.E., Bentorki A.A., Abat C., Rolain J.M. Molecular epidemiology and distribution of serotypes, genotypes, and antibiotic resistance genes of *Streptococcus agalactiae* clinical isolates from Guelma, Algeria and Marseille, France. Eur J Clin Microbiol Infect Dis 2015;34(12):2339–2348 10.1007/s10096-015-2487-6 Epub 2015 Sep 28. 26415872

[18] Gherardi G., Imperi M., Baldassarri L., et al Molecular epidemiology and distribution of serotypes, surface proteins, and antibiotic resistance among group B streptococci in Italy. J.Clin Microbiol. 2007; 45: 2909–2916 10.1128/JCM.00999-07 Epub 2007 Jul 18. 17634303 PMC2045288

[19] McGee L., Chochua S., Li Z., et al Multistate, population-based distributions of candidate vaccine targets, clonal complexes, and resistance features of invasive Group B Streptococci within the US: 2015–2017. Clin Infect Dis 2020 2 15;ciaa151. 10.1093/cid/ciaa151 Online ahead of print. 32060499 PMC8071603

[20] Morozumi M., Wajima T., Takata M., Iwata S., Ubukata K. Molecular characteristics of Group B streptococci isolated from adults with invasive 10.1128/JCM.01183-16 Epub 2016 Aug 24. 27558182 PMC5078545

[21] Lachenauer C.S., Kasper D.L., Shimada J., et al Serotypes VI and VII predominant amog group B streptococci isolated grom pregnant Japanese women. J Infect Dis 1999;179(4):1030–1033 10.1086/314666 10068604

[22] Eskandarian N., Ismail Z., Neela V., van Belkum A., Desa M.M., Amin Nordin S. Antimicrobial susceptibility profiles, serotype distribution and virulence determinants among invasive, non-invasive and colonizing *Streptococcus agalactiae* (group B streptococcus) from Malaysian patients. Eur J Clin Microbiol Infect Dis 2015;34(3):579–584 10.1007/s10096-014-2265-x Epub 2014 Oct 31. 25359580 PMC4356882

[23] Bisharat N., Jones N., Marchaim D., et al Population structure of group B Streptococcus from a low-incidence region for invasive neonatal disease. Open J Obstet and Gynecol 2005;151(6):1875–1881 10.1099/mic.0.27826-0 15941995

[24] Sefty H., Klivitsky A., Bromberg M., Dichtiar R., Ami MB., Shohat T., Glatman-Freedman A. Israel Obstetric Survey Group (IOSG). Isr J Health Policy Res. 2016; 15: 5–42 eCollection 2016. 10.1186/s13584-016-0103-6 PMC5109778 27879969

[25] Valkenburg-van den Berg A.W., Sprij A.J., Oostvogel P.M., et al Prevalence of colonisation with group B Streptococci in pregnant women of a multi-ethnic population in The Netherlands. Eur J Obstet Gynecol Reprod Biol. 2006 2 1;124(2):178–83. 10.1016/j.ejogrb.2005.06.007 Epub 2005 Jul 18… 16026920

[26] Huang J., Li S., Li L., Wang X., Yao Z., Ye X. Alarming regional differences in prevalence and antimicrobial susceptibility of group B streptococci in pregnant women: A systematic review and meta-analysis. J Glob Antimicrob Resist. 2016 12;7:169–177 10.1016/j.jgar.2016.08.010 Epub 2016 Nov 5. 27837713

[27] German L., Solt I., Bornstein J., Ben-Harush S., Ben-Elishai M., Weintraub Z. Is there an increase in the incidence of GBS carrier rates among pregnant women in northern Israel. Harefuah 2006 12;145(12):866–9, 944 17220021

[28] Drai-Hasid R., Calderon-Margalit R., Lev-Sagie A., et al Ritual immersion in a Mikveh is associated with increased risk of Group B Streptococcal carrier state in Israeli parturient women. Open J Obstet Gynecol 2015;05(14):769–774.

[29] Spitzer J., Hennessy E., Neville L. High Group A Streptococcal carriage in the Orthodox Jewish community of north Hackney. Br J Gen Pract. 2001 2;51(463):101–5. 11217620 PMC1313922

[30] Neemuchwala A., Teatero S., Liang L., Martin I., Demzcuk W., McGeer A., Fittipaldi N. Genetic diversity and antimicrobial drug resistance of serotype VI Group B Streptococcus, Canada. Emerg Infect Dis. 2018 10;24(10):1941–1942 10.3201/eid2410.171711 30226176 PMC6154151

[31] Wang P., Tong J. and et al Serotypes, antibiotic susceptibilities, and multi-locus sequence type profiles of *Streptococcus agalactiae* isolates circulating in Beijing, China. PloS One 2015;10(3). 10.1371/journal.pone.0120035 eCollection 2015. 25781346 PMC4363692

